# Effect of antioxidants supplementation on the quality of Beetal buck semen stored at 4°C

**DOI:** 10.14202/vetworld.2017.1184-1188

**Published:** 2017-10-05

**Authors:** Archana Sarangi, Pardeep Singh, Meenakshi Virmani, A. S. Yadav, Subhasish Sahu, H. M. Ajithakumar, Anuradha Kumari, A. P. Rath

**Affiliations:** 1 Division of Animal Physiology, ICAR-National Dairy Research Institute, Karnal, Haryana, India; 2 Department of Veterinary Physiology and Biochemistry, Lala Lajpat Rai University of Veterinary and Animal Sciences, Hisar - 125 004, Haryana, India; 3 Department of Animal Genetics and Breeding, Lala Lajpat Rai University of Veterinary and Animal Sciences, Hisar - 125 004, Haryana, India; 4 Department of Livestock Production and Management, Lala Lajpat Rai University of Veterinary and Animal Sciences, Hisar - 125 004, Haryana, India; 5 Department of Pathology, Lala Lajpat Rai University of Veterinary and Animal Sciences, Hisar - 125 004, Haryana, India

**Keywords:** Beetal buck, glutathione and liquid preservation, oxidative stress, semen, seminal parameters, Vitamin E

## Abstract

**Aim:**

An experiment was designed to evaluate the role of Vitamin E and glutathione in improving the seminal parameters during hypothermic storage of liquid semen at 4°C for 72 h.

**Materials and Methods:**

Thirty-six semen ejaculates were collected by artificial vagina from 6 bucks (Beetal) during the normal reproduction season (September to November) at weekly interval. The samples were centrifuged, and the seminal plasma was removed. The sperm pellet was diluted with Tris-based extender and divided into three groups. Group T1: Control samples without antioxidants, Group T2: Samples supplemented with tocopherol at 3 mM, and Group T3: Samples supplemented with glutathione at 1 mM. The samples were evaluated for progressive motility, percent liveability, percent abnormal spermatozoa, and acrosome integrity after liquid preservation for 0, 24, 48, and 72 h. The level of lipid peroxidation and antioxidant enzymes, namely, glutathione peroxidase (GPx) and superoxide dismutase (SOD) were estimated after liquid preservation for 0 and 72 h.

**Results:**

It was observed that, after storage of semen at 4°C up to 72 h, the progressive sperm motility, percent liveability, percent abnormal spermatozoa, and percent intact acrosomes were significantly (p<0.05) higher in group T2 and T3 samples as compared to control. However, the level of lipid peroxidation in T2 and T3 groups was significantly (p<0.05) lower after 72 h of incubation at 4°C. Similarly, GPx and SOD values were significantly (p<0.05) increased in T2 and T3 groups after 72 h of storage at 4°C.

**Conclusion:**

Thus, it can be concluded that Vitamin E and glutathione supplementation at 3 mM and 1 mM, respectively, while preserving the semen samples at 4°C helped in maintaining the seminal parameters up to 72 h and protected the spermatozoa from oxidative damage.

## Introduction

Goat husbandry is the backbone of rural economy in India as it helps to sustain the livelihood of rural poor because of providing meat, milk, skin, hair, and droppings. Goat meat (Chevon) is most preferred and widely consumed meat in the country, and goat milk has traditionally been known for its medicinal properties, and it’s all round health-promoting traits [1]. It is need of the hour to exploit the goat industry to its fullest to meet the gap between the market supply and demand.

For improvement of genetic program in goat, artificial insemination with liquid or frozen semen and quality of semen plays a major role for it. Sperm cells have a high content of polyunsaturated fatty acids (PUFAs) in their membranes, and they lack a significant cytoplasmic component containing antioxidants [2]. Therefore, they are susceptible to lipid peroxidation by the action of the reactive oxygen species (ROS) [3]. It results in the inhibition of both sperm adenosine triphosphate (ATP) production and sperm movement, particularly forward progression [4].

Antioxidants include non-enzymatic components such as Vitamin C, Vitamin E, selenium, and glutathione and a series of antioxidant enzymes such as glutathione peroxidase (GPx), catalase, and superoxide dismutase (SOD). Vitamin E (α-tocopherol) is the major chain breaking and lipid-soluble antioxidant that acts to support the cell defense mechanism. It readily donates the hydrogen from the hydroxyl (-OH) group on the ring structure to free radicals, making them unreactive. It is located primarily within the phospholipid bilayer of cell membranes and is effective in preventing lipid peroxidation, i.e., oxidative deterioration of PUFAs in sperm membrane. Glutathione can be biosynthesized in the body from amino acids cysteine, glutamic acid, and glycine. Addition of glutathione to the semen extenders may decrease or prevent the emergence of free radicals that can ruin the plasma membrane [5], thus reducing the loss of sperm motility, increasing the viability, plasma membrane integrity, and acrosomal integrity. The addition of Vitamin E and glutathione as primary antioxidants to the semen extender prevented the loss of sperm motility by inhibition of lipid peroxidation caused by ROS in chilled boar spermatozoa [6], frozen-thawed bull semen, and in goats also preserved all seminal parameters [7].

From the literature, it seems very little work that has been done on the addition of antioxidants in Beetal buck semen, therefore, it was considered to carry our studies on *in vitro* addition of glutathione and Vitamin E to the semen extender to maintain the keeping quality of buck semen.

## Materials and Methods

### Ethical approval

Permission of Animal Ethics Committee is not required to pursue such type of study. Semen samples were collected from well-maintained healthy animals for this study as per standard collection method.

### Location and climate

The experimental trial was carried out at the University goat farm, College of Veterinary Sciences, Lala Lajpat Rai University of Veterinary and Animal Sciences (Hisar, Haryana), which is situated in Trans-Gangetic Plain Region of India at longitude 75° 43′ 02.84″ E and the latitude of 29° 09′ 14.28″ N and at altitude of 234 m above mean sea level.

### Animals

The Beetal breed of goat is also considered as the very profitable breed to raise in the commercial goat farm. This breed seconds after Jamunapuri on the basis of milk production. The Beetal goat is a dual purpose (milch and meat) breed. Their skin is of very good quality for making leather goods which have demand in the market. The study was conducted with six Beetal goats, and they were housed in well-ventilated sheds which are maintained under proper hygienic conditions. Goats in the shed were exposed to a temperature range of 35-45°C (average 40°C) and relative humidity (RH) of around 15-20%. The animals were provided with clean water *ad libitum*.

### Experimental procedure

A total of 36 semen ejaculates were collected from six Beetal bucks at weekly interval with the help of artificial vagina. On day 0, ejaculates were collected and transferred to a water bath maintained at 37°C, seminal plasma was separated by centrifuging the semen at 1500 rpm for 10 min at 16°C, and tris-egg yolk extender was added to the semen pellet. The extended semen was divided into three parts, marked as T1 (control), T2 (semen supplemented with Vitamin E at 3 mM), and T3 (semen supplemented with glutathione at 1 mM). The samples were kept in liquid storage at 4°C for 72 h and evaluated for various morphological parameters, namely, progressive sperm motility, percent livability, percent acrosomal integrity, and percent abnormalities at 0, 24, 48, and 72 h of incubation, using the standard protocol [8]. The level of lipid peroxidation for malondialdehyde (MDA) was determined [9], and level of antioxidant enzymes, i.e., GPx and SOD were measured in the semen samples at 0 and 72 h of incubation, using the protocol [10,11], respectively.

### Statistical analysis

Data thus obtained were analyzed by the analysis of variance under complete randomized design using SPSS-20.0, a statistical package (SPSS Inc., Chicago, IL, USA) [12]. All data were presented as mean±standard error of the mean.

## Results

The percent progressive sperm motility, percent of live sperm, percent of abnormal spermatozoa, and percent intact acrosome (mean±SE) in liquid-preserved semen of Beetal bucks in control and both the treatment, i.e. after addition of Vitamin-E and glutathione at 4°C after 0, 24, 48, and 72 h of incubation are presented in Table-1.

**Table-1:** Percent seminal parameters (mean±SE) of buck spermatozoa preserved with antioxidants up to 72 h of incubation at 4°C.

Seminal parameters	Day 0			Day 1			Day 2			Day 3		
			
	T1	T2	T3	T1	T2	T3	T1	T2	T3	T1	T2	T3
% progressive sperm motility	55.83^b^±1.04	63.33^a^±1.02	64.31^a^±1.06	45.14^b^±1.04	57.36a±1.10	57.36^a^±0.92	33.61^b^±1.51	50.83^a^±0.88	50.81^a^±0.81	15.69^b^±0.94	43.75^a^±0.70	42.78^a^±0.58
% live sperms	59.60^b^±1.08	67.51^a^±1.12	68.11^a^±1.03	48.63^b^±1.20	61.31a±1.10	61.03^a^±1.00	36.86^b^±1.65	54.82^a^±0.99	54.65^a^±0.98	18.64^b^±1.08	47.05^a^±0.74	46.28^a^±0.61
% abnormal sperms	12.21^b^±0.22	11.11^a^±0.20	10.73^a^±0.20	12.34^b^±0.27	10.43a±0.19	10.02^a^±0.18	12.42^b^±0.30	9.63^a^±0.18	9.25^a^±0.16	12.65^b^±0.34	8.74^a^±0.16	8.41^a^±0.12
% intact acrosome	57.54^b^±1.04	65.38^a^±1.04	66.41^a^±1.07	46.78^b^±1.06	58.94a±1.15	59.34^a^±0.93	35.23^b^±1.53	52.49^a^±0.92	52.38^a^±0.82	16.99^b^±0.88	45.11^a^±0.75	44.64^a^±0.60

Different superscripts (a and b) within the row for a particular day differ significantly (p<0.05). Group T1: Control. Group T2: Supplemented with Vitamin E at 3 mM. Group T3: Supplemented with glutathione at 1 mM. SE=Standard error

The mean progressive sperm motility of control semen samples (T1) at 0, 24, 48, and 72 h differed significantly (p<0.05) as compared to T2 group supplemented with Vitamin E at 3 mM and T3 group supplemented with glutathione at 1 mM. There was a significant (p<0.05) increase in sperm motility at each observation for T2 and T3 than T1, and the increase was linear.

At 0 h of incubation, it was found that the percent live spermatozoa in semen were significantly higher (67.51±1.12) after addition of Vitamin E at a concentration of 3 mM and (68.11±1.03) after addition of glutathione at a concentration of 1 mM in tris-extended semen, as compared to control (59.60±1.08). In this study, there was a significant increase (p<0.05) in percent live spermatozoa and decrease in percent abnormal spermatozoa at 0 h of incubation in comparison to control semen samples. There was a decrease in percent abnormal spermatozoa in T2 and T3 groups that might be due to the protective effect of Vitamin E and glutathione on the sperm membrane; however, no report is available in Beetal buck semen to substantiate this finding. The percent spermatozoa with intact acrosome in Beetal buck semen at 0, 24, 48, and 72 h of incubation at 4°C were significantly higher (p<0.05) in T2 and T3 as compared to T1.

The level of oxidative stress parameters (lipid peroxidation, GPx, and SOD) in liquid-preserved semen of Beetal bucks in control and after addition of Vitamin-E and glutathione at 4°C after 0 and 72 h of incubation are presented in Table-2. MDA level of semen samples treated with Vitamin E and glutathione at the concentration of 3 mM and 1 mM, respectively, was comparable with control values at 0 h of incubation. However, at 72 h incubation, MDA level showed a significant decrease (p<0.05) in T2 group (2.65±0.06) and T3 group (2.56±0.05) in comparison to control group (2.88±0.06). GPx values in T2 and T3 were significantly (p<0.05) higher in comparison to T1 after 0 and 72 h of incubation. SOD values in Beetal buck semen were significantly higher in T2 and T3 groups ([3.50±0.41] and [3.40±0.27]) as compared to T1 (2.55±0.17), after 72 h of incubation though both groups showed statistically similar values at 0 h of incubation.

**Table-2:** Level of oxidative stress parameters (mean±SE) in spermatozoa incubated with antioxidants up to 72 h of incubation at 4°C.

Oxidative stress parameters	Day 0			Day 3		
	
	T1	T2	T3	T1	T2	T3
Lipid peroxidation (MDA in μg/ml)	2.48^b^±0.08	2.47^b^±0.06	2.38^b^±0.08	2.88^b^±0.06	2.65^a^±0.06	2.56^a^±0.05
GPx	23.38^b^±2.16	35.66^a^±1.94	36.2^a^±1.67	24.68^b^±1.78	45.39^a^±1.94	47.60^a^±2.06
SOD	2.87^b^±0.25	3.29^b^±0.34	3.66^b^±0.36	2.55^b^±0.17	3.50^a^±0.41	3.40^a^±0.27

Different superscripts (a and b) within the row are differ significantly (p<0.05). Group T1: Control. Group T2: Supplemented with Vitamin E at 3 mM. Group T3: Supplemented with glutathione at 1 mM. GPx=Glutathione peroxidase, SOD=Superoxide dismutase, MDA=Malondialdehyde, SE=Standard error

## Discussion

The effect of Vitamin E and glutathione on progressive sperm motility has not been studied so far in Beetal buck semen stored at 4°C; although few reports in ram semen have been reported. Zeitoun and Al-Damegh [13] observed better motility with 5 IU Vitamin E and 1 mM glutathione in ram semen after 96 h of incubation at 5°C in the refrigerator. Motility of semen samples containing Vitamin E and glutathione in our study was maintained higher than control up to 72 h of incubation at 4°C. This is also in consonance with the earlier studies where α-tocopherol at 4.5 mM was supplemented in Sirohi buck semen [14], and Vitamin E (1 mM) was supplemented in Alpine buck semen [15]. The significant role of Vitamin E was reported in maintenance and sustaining the quality of frozen-thawed spermatozoa including motility [15].

Supplementation of antioxidants (α-tocopherol) at 4.5 mM in Sirohi goat semen extender increased the duration of activity of spermatozoa during freeze-thawing where Saraswat *et al*. [14] reported that percent live sperms were significantly (p<0.05) higher in semen samples in which 7 mM/ml reduced glutathione and 9 mM/ml of ascorbic acid were added as compared to control. The results of the present study are in agreement with Saraswat *et al*. [14], who reported the least damaged acrosome during freezing after addition of α-tocopherol at 4.5 mM in Sirohi goat semen as compared to control, suggesting that addition of Vitamin E and glutathione has an important role in maintaining and sustaining the quality of buck semen. However, more percent live spermatozoa were observed at a concentration of 5 IU/ml and 1 mM/ml of Vitamin E and glutathione, respectively, in chilled-stored ram semen [13].

Percent abnormal spermatozoa decreased in the present study in the treated groups. This might be due to the reason that the supplementation of antioxidants may have reduced the generation of free radicals. Spermatozoa are susceptible to LPO during cryopreservation and thawing [16], which confers considerable mechanical stress on the cell membrane [17]. It has been noted in humans that ROS level has a positive correlation with the extent of apoptotic sperms [18]. Memon *et al*. [19] studied that addition of antioxidants, i.e., ascorbic acid (8.5 mg/ml), butylated hydroxytoluene (2 mM), cysteine (5 mM), and hypotaurine (10 mM) significantly reduced the amount of ROS in frozen-thawed spermatozoa with reduction in MDA level. It is, therefore, evident that the supplementation of antioxidants in the extending media reduces the production of ROS during freeze-thawing, thus protecting the spermatozoa from cryopreservation damage. Bansal and Bilaspuri [20] also reported that supplementation of 2 mM Vitamin E in cattle spermatozoa was a most effective antioxidant that reduced the LPO caused by reactive oxidative species and improved sperm motility and viability *in vitro* under induced oxidative stress. Our results were also supported by Zeitoun and Al-Damegh [13], who reported that MDA concentration was highest (p<0.05) in control than the ram semen treated with Vitamin E (1, 5, and 10 IU), cysteine (1, 5, and 10 mM), or glutathione (0.5, 1, and 2 mM) at 5°C.

Vitamin E and GSH in conjunction with GPx prevent and restrict the production and propagation of reactive free radicals in avian semen [21]. GPx values increased significantly in T2 and T3 groups in the present study after 0 and 72 h of incubation. The level of SOD was also significantly higher in supplemented groups, after 72 h of incubation though both groups showed statistically similar values at 0 h of incubation. These findings can be substantiated by the report of Zeitoun and Al-Damegh [13], who reported that GPx and SOD values significantly increased in diluents containing 5 IU Vitamin E and 1 mM and 2 mM glutathione within sperm cells and seminal plasma.

## Conclusion

The study revealed that supplementation of antioxidants in the form of Vitamin E at 3 mM and glutathione at 1 mM in tris extender helped in liquid preservation of buck semen up to 72 h at 4°C in refrigerator with higher progressive sperm motility, higher liveability percentage, and higher percentage of sperm with intact acrosome, and this also led to decreased lipid peroxidation and increase in antioxidant enzymes, thus offering protection to the spermatozoa from the free radicals generated during storage of semen.

## Authors’ Contributions

AS carried out the experiment which analyzed the data and drafted the manuscript. PS and MV designed the experiment and also supervised the laboratory work. ASY provided the animals from his farm and contributed to statistical analysis. SS and AK helped to draft the manuscript. HMA and APR reviewed the manuscript efficiently. All authors read and approved the final manuscript.
